# 753 Segregation, Vulnerability, and Fundamental Causes: Understanding the Social Dimensions of Burn Injury

**DOI:** 10.1093/jbcr/irac012.306

**Published:** 2022-03-23

**Authors:** Colette Ngana

**Affiliations:** Case Western Reserve University, Cleveland Heights, Ohio

## Abstract

**Introduction:**

Sociologists have long investigated the enduring link between place and health. Despite prolific research in this field, there are health issues that remain obscured in the literature. The goal of this work is to examine the spatial-health relationship(s) between place of residence and burn injuries, the socially produced vulnerability of individuals living in high-risk areas, and the experience of being burned in these identified areas. Fundamental cause theory, which focuses on an individual’s ability to either avoid health risks or minimize the consequences of these risks, is employed to demonstrate the worsening effects of socioeconomic status (classism) and race (racism) on burn injuries. Managing health risks is dependent upon access to and utilization of flexible resources (knowledge, money, freedom, power, prestige, and social networks) that are differentially distributed across populations based on social conditions like age, race-ethnicity, socioeconomic status, and neighborhood infrastructure.

**Methods:**

Exploratory spatial and cluster analysis using ArcGIS (a geographic information system) of accidental, at-home burn incidence in an historically redlined county.

**Results:**

Analysis shows burn injuries tend to cluster in areas marked by low-income, higher numbers of racial-ethnic minorities, and poorer quality housing. Table 1 shows preliminary descriptive data about the study population.

**Conclusions:**

Despite advances in treatment and prevention, there are socially-constructed and salient environmental risks that leave some communities vulnerable to burn injury. Historical practices, like racial residential segregation and redlining, have concentrated racial-ethnic minority communities marked by lower-income, poorer quality housing, and other burn-risk factors.

